# Inhibition of Protein Tyrosine Phosphatase Improves Angiogenesis via Enhancing Ang-1/Tie-2 Signaling in Diabetes

**DOI:** 10.1155/2012/836759

**Published:** 2012-02-12

**Authors:** Jian-Xiong Chen, Qinhui Tuo, Duan-Fang Liao, Heng Zeng

**Affiliations:** ^1^Department of Pharmacology and Toxicology, University of Mississippi Medical Center, 2500 North State Street, Jackson, MS 39216, USA; ^2^Department of Pharmacology, University of South of China, Hengyang 421001, China; ^3^Department of Traditional Chinese Diagnostics, School of Pharmacy, Hunan University of Chinese Medicine, Hunan, Changsha 410208, China

## Abstract

Diabetes is associated with impairment of angiogenesis such as reduction of myocardial capillary formation. Our previous studies demonstrate that disruption of Angiopoietin-1 (Ang-1)/Tie-2 signaling pathway contributes to the diabetes-associated impairment of angiogenesis. Protein tyrosine phosphatase (PTP) has a critical role in the regulation of insulin signal by inhibition of tyrosine kinase phosphorylation. In present study, we examined the role of protein tyrosine phosphatase-1 (SHP-1) in diabetes-associated impairment of Ang-1/Tie-2 angiogenic signaling and angiogenesis. SHP-1 expression was significantly increased in diabetic db/db mouse hearts. Furthermore, SHP-1 bond to Tie-2 receptor and stimulation with Ang-1 led to SHP-1 dissociation from Tie-2 in mouse heart microvascular endothelial cell (MHMEC). Exposure of MHMEC to high glucose (HG, 30 mmol/L) increased SHP-1/Tie-2 association accompanied by a significant reduction of Tie-2 phosphorylation. Exposure of MHMEC to HG also blunted Ang-1-mediated SHP-1/Tie-2 dissociation. Knockdown of SHP-1 significantly attenuated HG-induced caspase-3 activation and apoptosis in MHMEC. Treatment with PTP inhibitors restored Ang-1-induced Akt/eNOS phosphorylation and angiogenesis. Our data implicate a critical role of SHP-1 in diabetes-associated vascular complications, and that upregulation of Ang-1/Tie-2 signaling by targeting SHP-1 should be considered as a new therapeutic strategy for the treatment of diabetes-associated impairment of angiogenesis.

## 1. Introduction

 Angiogenesis is mainly regulated by the vascular endothelial growth factor (VEGF)/VEGF receptor (VEGFR) and the angiopoietins/Tie-2 system. Receptor tyrosine kinases (RTKs) represent a major class of cell-surface molecules that regulate angiogenesis. VEGFR and the Tie-2 receptor are the principal RTK families and play critical roles in the regulation of angiogenesis [1]. Impaired angiogenesis leading to microvascular insufficiency represents a major cause of end-stage organ failure among diabetics. The underlying molecular mechanisms, however, are poorly understood [2, 3]. Myocardial angiogenesis is significantly impaired in patients with diabetes mellitus which may contribute to the high mortality after myocardial infarction [4, 5]. So far, few studies have focused on the identification of factors that affect myocardial angiogenesis in the setting of diabetes. A previous study showed that VEGF-induced migration and VEGFR-mediated signal transduction were severely impaired in the monocytes of diabetic patients [6, 7]. Further, VEGFR expression was significantly reduced in the heart of diabetic patients compared with nondiabetic individuals. This was accompanied by an impairment of VEGFR phosphorylation, suggesting that decreased VEGF expression and defective VEGF signaling may play a key role in the diabetes-associated impairment of angiogenesis [8]. Our previous studies have found that defective RTK signaling transduction is not only limited to VEGF/VEGFR, but is also associated with the disruption of Ang-1/Tie-2 angiogenic signaling and angiogenesis under hyperglycemic conditions and in diabetes [9–11].

 Protein tyrosine phosphatase (PTP) has been shown to negatively regulate insulin signaling by dephosphorylation of insulin receptor tyrosine kinase [12, 13]. PTP also has a critical role in the regulation of growth factors signal transduction by de-phosphorylation of RTK. PTP inhibition has been shown to promote collateral growth and enhance VEGF-induced angiogenesis in a rat model of hindlimb ischemia [14, 15]. The cytoplasmic protein tyrosine phosphatase-1 (SHP-1) expresses primarily in hematopoietic lineages and endothelial cells [16–19] and negatively regulates growth factor receptors phosphorylation [17, 18, 20, 21]. SHP-1 expression is upregulated as a result of abnormal inflammatory responses in diabetes patients [22]. A previous study revealed that Tie-2 receptor was the substrates for tyrosine phosphatase-2 (SHP-2) [23]. To date, little is known of the functional role of SHP-1 on the Ang-1/Tie-2 signaling and impairment of angiogenesis in diabetes.

In our present study, we hypothesize that hyperglycemia and diabetes impair Ang-1/Tie-2 signaling and angiogenesis by a mechanism involving upregulation of SHP-1 expression and SHP-1/Tie-2 interaction. Our data suggest that increased SHP-1 has a crucial role in the diabetes-associated impairment of angiogenesis by interfering with the Ang-1/Tie-2 angiogenic signaling.

## 2. Materials and Methods

### 2.1. Mouse Heart Microvascular Endothelial Cells (MHMECs)

MHMECs was isolated from C57BL/6J mouse hearts and cultured as previously described [24–26]. Primary cultures of MHMEC, between passages 4 and 10, were used in all experiments.

### 2.2. Endothelial Cell Apoptosis and Caspase-3 Activity

To induce apoptosis, MHMEC were exposed to serum-free medium for 72 hours under high glucose (HG, 30 mmol/L) or normal glucose (NG, 5 mmol/L) conditions. Endothelial cell apoptosis was measured by counting TUNEL positive cells per 100 endothelial cells following the manufacturer's instructions (Promega, WI). Caspase-3 activity was measured using the caspase-3 kit (Sigma, MO).

### 2.3. Immunoprecipitation of Tie-2 and Blotting with SHP-1 or Phospho-Tyrosine

 MHMEC lysates were immunoprecipitated with anti-mouseTie-2 antibody followed by incubation with a 1 : 1 protein A: protein G-sepharose slurry. The immunoprecipitates were then subjected to SDS-PAGE gels and transferred to nitrocellulose membranes. The membranes were immunoblotting anti-SHP-1 (1 : 1000, Santa Cruz, CA) or anti-phospho-tyrosine (4G10, 1 : 1000 Upstate Biotech, NY). The membranes were washed and incubated with a secondary antibody coupled to horseradish peroxidase.

### 2.4. SHP-1, Tie-2, Akt, and eNOS Expression

 Fifty micrograms of total protein of myocardial tissue or MHMEC lysates were separated using SDS-gel electrophoresis. The membranes were immunoblotted with SHP-1 (1 : 1000), eNOS and Tie-2 (1 : 1000, Cell Signaling Technology, MA) antibodies. For eNOS and Akt phosphorylation, the membranes were immunoblotted with rabbit anti-phospho-Akt and anti-phospho-eNOS (1 : 1000, Cell Signaling, MA). *β*-Actin was used as a loading control (1 : 1000, Cell Signaling Technology, MA) on the same nitrocellulose blots after stripping. The membranes were washed and incubated with a secondary antibody coupled to horseradish peroxidase, and densitometric analysis was carried out using image acquisition and analysis software (TINA 2.0).

### 2.5. SHP-1 siRNA Transfection

MHMEC (approx. 80% confluent) was treated with SHP-1 siRNA (mouse, Santa Cruz, CA) for 24 hours to inhibition of SHP-1 expression according to the manufacturer's instructions. Knockdown of SHP-1 was confirmed by Western blot analysis of SHP-1 protein expression (data not shown).

### 2.6. Measurement of MHMEC Survival by MTT Assay

 Cell survival was assayed using the MTT assay kit (Roche Diagnostic Corp., Indianapolis, IN).

### 2.7. Systemic Delivery of a PTP Inhibitor in Diabetic db/db Mice

The C57BL/6J mice and db/db mice were purchased from Jackson Laboratory (Bar Harbor, Maine). Sixteen male db/db mice at 12 weeks of age were divided into two groups: [1] the PTP inhibitor treatment group: db/db mice (*n* = 8) received oral bioavailable organovanadium compound, bis-(maltolato)oxovanadium (IV) (BMOV, 0.2 g/L) in their drinking water for 2 weeks; [2] the db/db control group received drinking water alone for 2 weeks. All procedures were in compliance with the Institute for Laboratory Animal Research Guide for the Care and Use of Laboratory Animals and were approved by the University of Mississippi Medical Center Institutional Animal Care and Use Committee.

### 2.8. *In Vivo* Myocardial Capillary Density Analysis

The experimental mouse hearts were harvested and immediately flash frozen. Tissue sections were incubated with fluorescein-labeled antibodies against Griffonia Bandeiraea Simplicifolia Isolectin B4 (IB4, 1 : 200, Sigma Co) to label the endothelial cells, and myocardial capillary density was measured using image analysis software (Image J, NIH). The number of capillaries (IB4) was counted and expressed as capillary density per mm^2^ [9–11].

### 2.9. *Ex Vivo* Angiogenesis Assay

 Mouse aortae were isolated and collected from C57BL/6J and db/db mice, placed in the middle of organ culture dishes and overlaid with 300 *μ*L of ECM gel (Sigma Co, MO). After solidification, the ECM gel was covered with 10% FBS EGM in the presence or absence of recombinant human Ang-1 (250 ng/mL). Vessel outgrowth at day 5 was examined using a Nikon TE-300 microscope. The area of vessel outgrowth was quantified using image acquisition and analysis software (Image J, NIH) [9, 11, 25, 26].

### 2.10. Statistical Analysis

All results were expressed as mean ± SD. Statistical analysis was performed using unpaired student *t*-test. A *P* value < 0.05 denoted significance.

## 3. Results

### 3.1. SHP-1 Expression Is Upregulated in the Diabetic db/db Mouse Hearts

**Figure 1:**
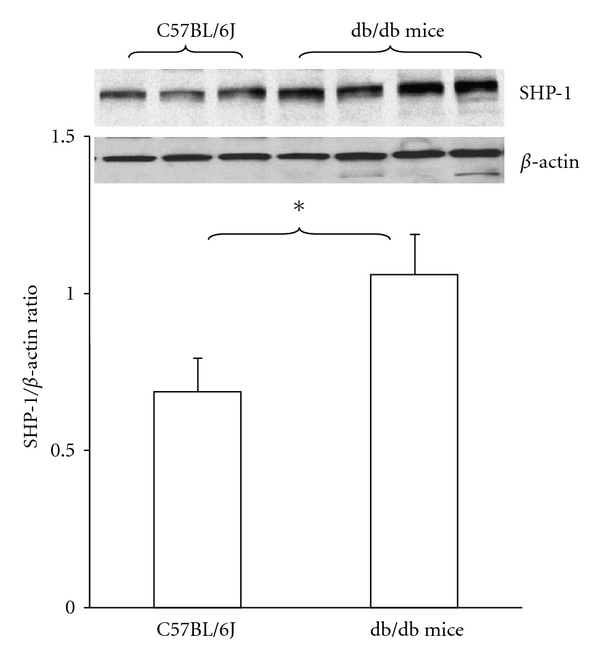
Expression of SHP-1 in the control C57BL/6J and db/db mouse hearts. Western blot showing SHP-1 expression in control C57BL/6J and diabetic db/db mouse hearts. Densitometric data shows that SHP-1 protein expression was significantly increased in diabetic db/db mouse hearts in comparison with control C57BL/6J mice (*n* = 3-4 mice, *P < 0.05).

Western blot analysis showed that SHP-1 protein was expressed both in C57BL/6J mouse and diabetic db/db mouse hearts. Intriguingly, the expression of SHP-1 protein was significantly increased in db/db mouse hearts in comparison to C57BL/6J controls (Figure 1). The SHP-2 protein expression was unchanged in db/db mouse hearts in comparison to C57BL/6J controls (data not shown).

### 3.2. HG Increases SHP-1/Tie-2 Association and Decreases Tie-2 Tyrosine Phosphorylation in MHMEC

**Figure 2:**
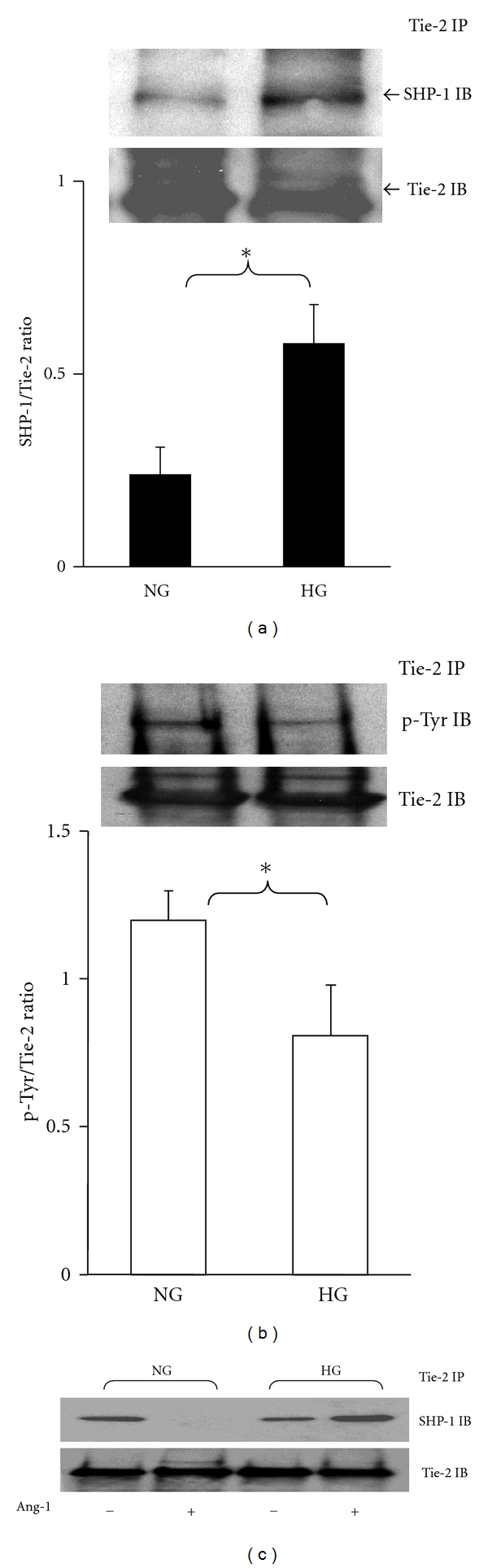
Immunoprecipitation and Western blot analysis showing that SHP-1 associates with Tie-2 receptor and high glucose alters the SHP-1/Tie-2 association in MHMEC. (a) SHP-1 binds to the Tie-2 receptor under normal glucose (NG) conditions; exposure of MHMEC to high glucose (HG) leads to a significant increase in the SHP-1/Tie-2 association (*n* = 4 cell lines, *P < 0.05). (b) Exposure of MHMEC to high glucose (HG) conditions results in a significant reduction of Tie-2 phosphorylation (*n* = 3 cell lines, *P < 0.05). (c) Under normal glucose (NG) conditions, stimulation of MHMEC with Ang-1 (250 ng/mL) for 15 minutes leads to a dissociation of SHP-1 from Tie-2. Under HG conditions, Ang-1 failed to induce SHP-1/Tie-2 dissociation. These results represent three different cell lines and experiments.

To examine whether SHP-1 binds to Tie-2, MHMEC lysates were immunoprecipitated with Tie-2 antibody and blotted with SHP-1 antibody. As shown in Figure 2(a), SHP-1 bond to Tie-2 and formed a Tie-2/SHP-1 complex. Exposure of MHMEC to HG (30 mmol/L) resulted in a significant increase in SHP-1/Tie-2 association. This was accompanied by a significant decrease in Tie-2 tyrosine phosphorylation (Figure 2(b)).

### 3.3. Ang-1 Induces SHP-1 Dissociation from Tie-2 and This Effect Is Ameliorated by HG in MHMEC

To determine whether SHP-1 was involved in Ang-1-mediated Tie-2 activation, the effect of Ang-1 on Tie-2/SHP-1 association was examined. As shown in Figure 2(c), stimulation of MHMEC with Ang-1 (250 ng/mL) resulted in a dissociation of SHP-1 from Tie-2 receptor. Ang-1 failed to cause SHP-1dissociation from Tie-2 under HG conditions (Figure 2(c)).

### 3.4. SHP-1 siRNA Attenuates HG-Induced Caspase-3 Activation and Apoptosis in MHMEC

**Figure 3:**
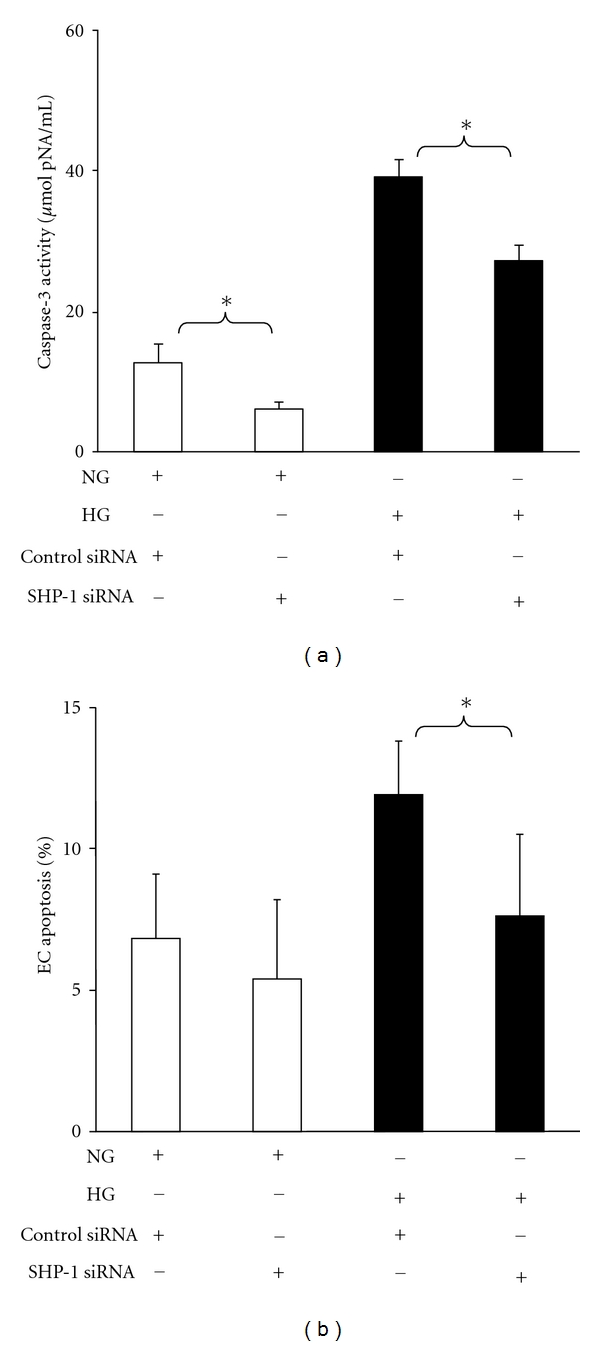
Knockdown of SHP-1 by siRNA blunts HG-induced cell apoptosis in MHMEC. (a) Caspase-3 ELISA analysis showing that transfection of MHMEC with SHP-1 siRNA significantly attenuated caspase-3 activation under NG or HG conditions (*n* = 3 cell lines, *P < 0.05). (b) TUNEL staining showing that MHMEC transfected with SHP-1 siRNA significantly inhibited high glucose-induced endothelial cell apoptosis (*n* = 3 cell lines, *P < 0.05).

Next, the functional role of SHP-1 in high glucose-induced endothelial dysfunction was investigated. Treatment of MHMEC with SHP-1 siRNA significantly suppressed caspase-3 activity under normal glucose (NG) and HG conditions (Figure 3(a)). Further, treatment of MHMEC with SHP-1 siRNA significantly blunted HG-induced endothelial cell apoptosis (Figure 3(b)).

### 3.5. Inhibition of PTP Promotes Ang-1-Induced Angiogenic Signaling under HG Conditions

**Figure 4:**
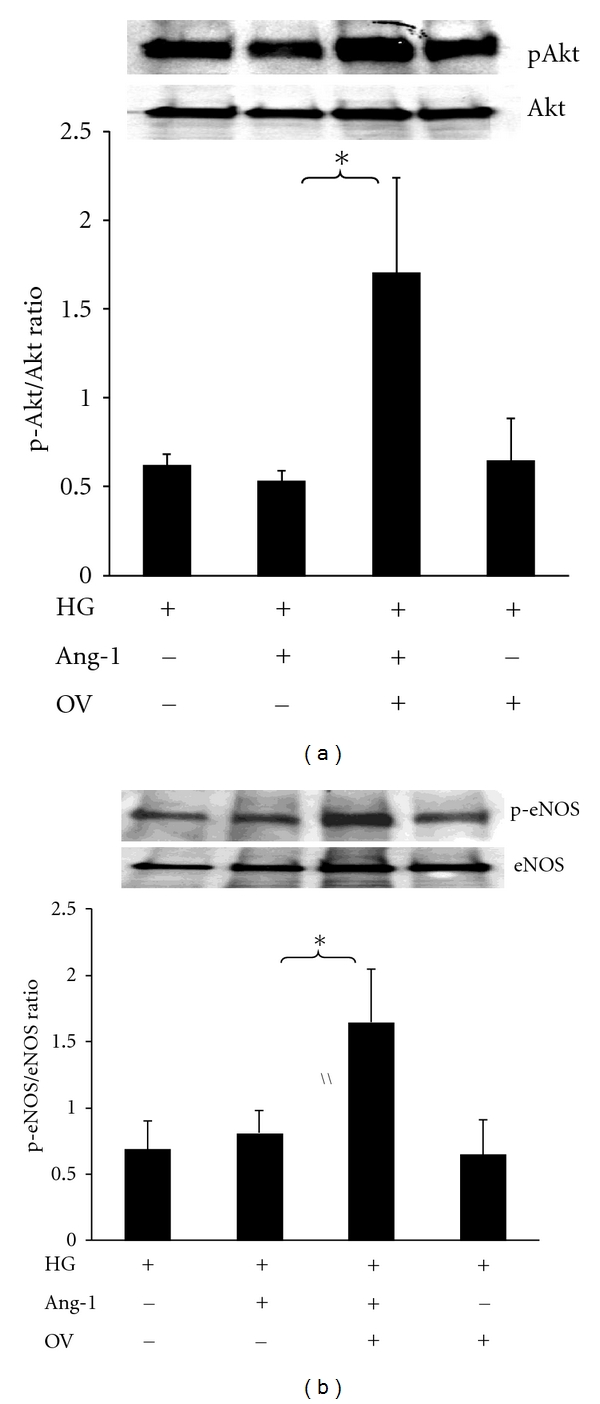
Inhibition of PTP enhances Ang-1-induced Akt and eNOS phosphorylation under HG conditions. (a) Western blot showing that pretreatment of MHMEC with the PTP inhibitor orthovanadate (OV, 5 *μ*M) for 30 minutes leads to an increase in Akt phosphorylation in response to Ang-1 (250 ng/mL) stimulation under high glucose conditions (*n* = 3, *P < 0.05). (b) Western blot showing that pretreatment of MHMEC with the PTP inhibitor OV for 30 minutes results in a significant increase in Ang-1-induced eNOS phosphorylation under high glucose conditions (*n* = 3, *P < 0.05).

To further investigate the role of PTP in the Ang-1/Tie-2 signaling, the PTP inhibitor on Ang-1-induced Akt/eNOS phosphorylation was examined in MHMEC under HG conditions. MHMEC was pretreated with PTP inhibitor sodium orthovanadate (OV, 5 *μ*M) for 30 minutes, followed by Ang-1 (250 ng/mL) treatment for 15 minutes. Exposure of MHMEC to OV significantly enhanced Ang-1-induced Akt and eNOS phosphorylation under HG conditions. Pretreatment of MHMEC with OV alone had no significant effect on Akt and eNOS phosphorylation under HG conditions (Figures 4(b) and 4(b)).

### 3.6. Inhibition of PTP Enhances Ang-1-Mediated Cell Survival in MHMEC

**Figure 5:**
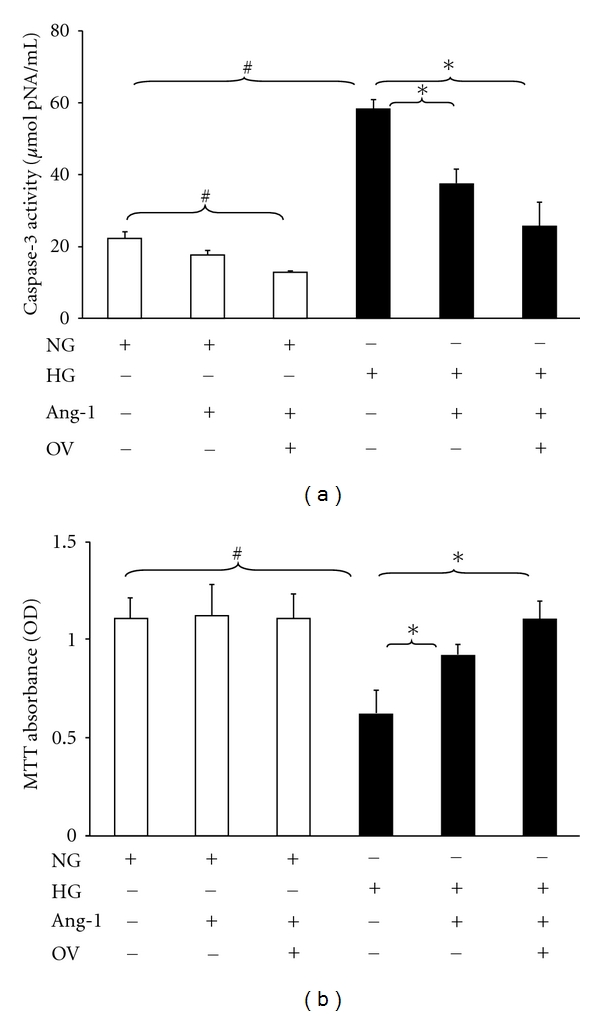
Inhibition of PTP promotes Ang-1-induced suppression of caspase-3 activation and increases cell survival under HG conditions. (a) Caspase-3 activity assay showing that exposure of MHMEC to Ang-1 significantly attenuates HG-induced caspase-3 activation; pretreatment of MHMEC with OV before the Ang-1 treatment further enhances the effect of Ang-1 on caspase-3 activity under HG conditions (*n* = 3–5 cell lines, ^#^ *P* < 0.05 versus NG, *P < 0.05 versus HG). (b) MTT assay for the cell survival showing that pretreatment with Ang-1 increases endothelial cell survival; pretreatment with OV before the Ang-1 treatment further enhances the effect of Ang-1 on endothelial cell survival under HG conditions (*n* = 3–5 cell lines, ^#^ *P* < 0.05 versus NG, *P < 0.05 versus HG).

Treatment of MHMEC with Ang-1 (250 ng/mL) significantly attenuated caspase-3 activity. The inhibitory effect of Ang-1 on caspase-3 activity was further enhanced in the presence of OV (Figure 5(a)). Ang-1 has a critical role in the regulation of endothelial cell survival. Treatment of MHMEC with Ang-1 (250 ng/mL) also increased cell survival under HG conditions. Similarly, Ang-1-induced cell survival was greatly enhanced in the presence of OV under HG conditions. Surprisingly, OV alone had little effect on cell survival (Figure 5(b)).

### 3.7. Inhibition of PTP Augments Ang-1-Induced Vessel Outgrowth in db/db Mouse

**Figure 6:**
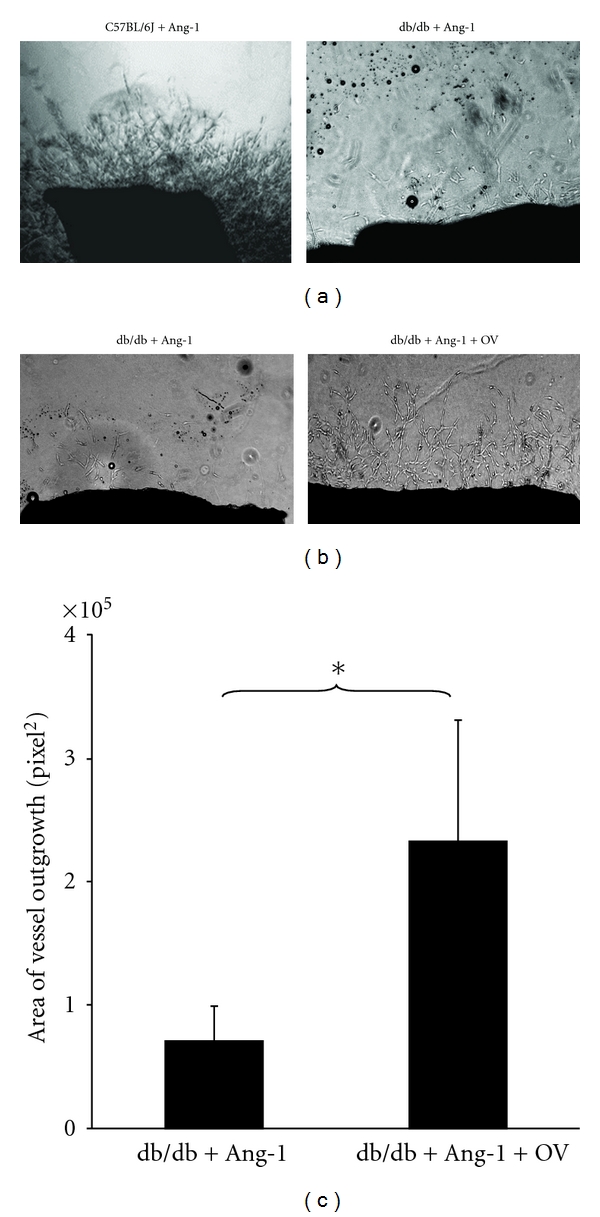
Treatment with PTP inhibitor restores the angiogenic response to Ang-1 in diabetic db/db mouse. (a) Representative images of Ang-1-induced aortic explants sprouting in C57BL/6J and diabetic db/db mice. Stimulation of C57BL/6J mouse aortic explants with Ang-1 (250 ng/mL) leads to robust vessel outgrowth. Ang-1-induced vessel outgrowth was blunted in the db/db mouse aortic explants. (b) Representative images of Ang-1-induced vessel outgrowth in the db/db mouse aortic explants in the presence and absence of PTP inhibitor OV. (c) Quantitative areas of vessel outgrowth in db/db mouse aortic explants showing Ang-1-induced vessel outgrowth were significantly increased in the presence of PTP inhibitor OV (*n* = 4–8 mice, *P < 0.05).

As shown in Figure 6(a), exposure of C57BL/6J aortic explants to Ang-1 (250 ng/mL) resulted in a robust angiogenic response. In contrast, the Ang-1-induced vessel outgrowth was greatly diminished in db/db mouse vessel explants compared with C57BL/6J mouse. In the presence of OV, Ang-1-induced vessel outgrowth was significantly augmented compared to the db/db control group (Figures 6(b) and 6(c)).

### 3.8. Inhibition of PTP Increases eNOS Expression and Capillary Density in db/db Mouse Hearts

**Figure 7:**
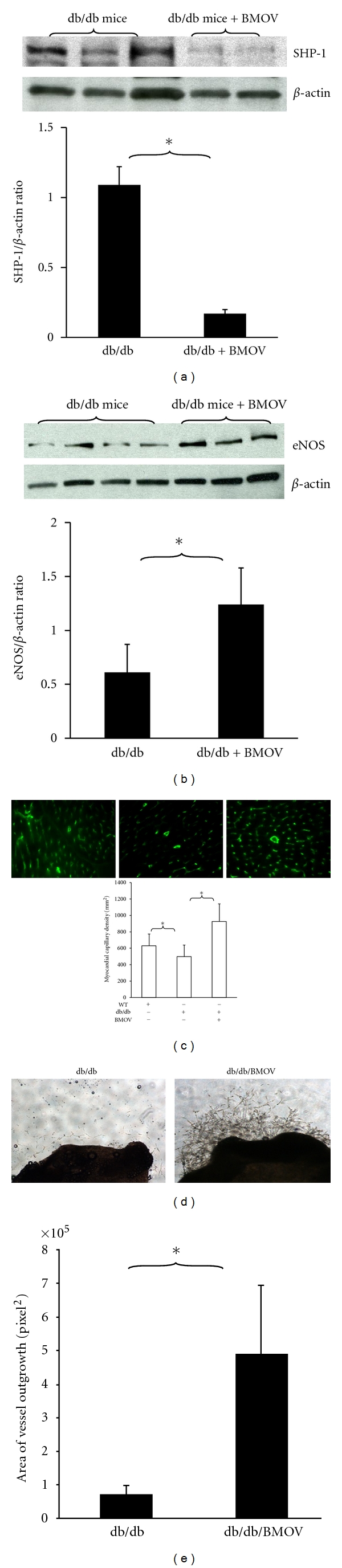
Systemic treatment with the PTP inhibitor attenuates SHP-1 expression and increases eNOS and myocardial capillary density in db/db mice. (a) Western blot analysis showing that treatment with BMOV for 2 weeks leads to a significant suppression of SHP-1 expression in the db/db mice compared to db/db control without BMOV (*n* = 3 mice, *P < 0.05). (b) Western blot analysis showing that treatment with BMOV for 2 weeks results in a significant increase in eNOS expression in the db/db mice compared to db/db control (*n* = 3-4 mice, *P < 0.05). (c) Myocardial capillary density was significantly increased in the BMOV-treated db/db mice compared to db/db control with BMOV (*n* = 8, *P < 0.05). (d) Quantitation of areas of vessel outgrowth showing Ang-1-induced vessel explants sprouting is significantly improved in the BMOV-treated db/db mice compared to db/db control group (*n* = 4–8 mice, *P < 0.05).

To examine whether inhibition of PTP augments myocardial angiogenesis in diabetic hearts, an orally bioavailable PTP inhibitor bis-(maltolato)oxovanadium (IV) (BMOV) was given to the experimental db/db mice. Treatment of db/db mice with BMOV for 2 weeks resulted in a significant decrease in SHP-1 expression (Figure 7(a)). This was accompanied by a significant increase in eNOS expression in db/db mouse hearts (Figure 7(b)). Immunohistochemical studies revealed that myocardial capillary density was significantly decreased in db/db mouse hearts when compared to C57BL/6J controls. Myocardial capillary density was significantly increased in the BMOV-treated db/db mice (Figure 7(c)). Our *ex vivo* angiogenesis studies also showed that Ang-1-induced vessel outgrowth was significantly improved in the BMOV-treated db/db mice in comparison with db/db control group (Figure 7(d)). 

## 4. Discussion

 The current study demonstrates that SHP-1 binds to the Tie-2 to form a SHP-1/Tie-2 complex and that the Ang-1, an agonist of Tie-2, causes SHP-1 dissociation from Tie-2. This finding implicates a potential role of SHP-1 in Ang-1-induced Tie-2 phosphorylation. Intriguingly, high glucose increases formation of the SHP-1/Tie-2 complex and this is accompanied by Tie-2 dephosphorylation. Ang-1 failed to cause SHP-1 dissociation from Tie-2 under HG conditions. Suppression of SHP-1 expression significantly attenuated endothelial apoptosis and improved diabetes-associated impairment of angiogenesis. These data strongly suggest a critical role for SHP-1 and the SHP-1/Tie-2 association in diabetes-associated impairment of angiogenesis.

**Figure 8:**
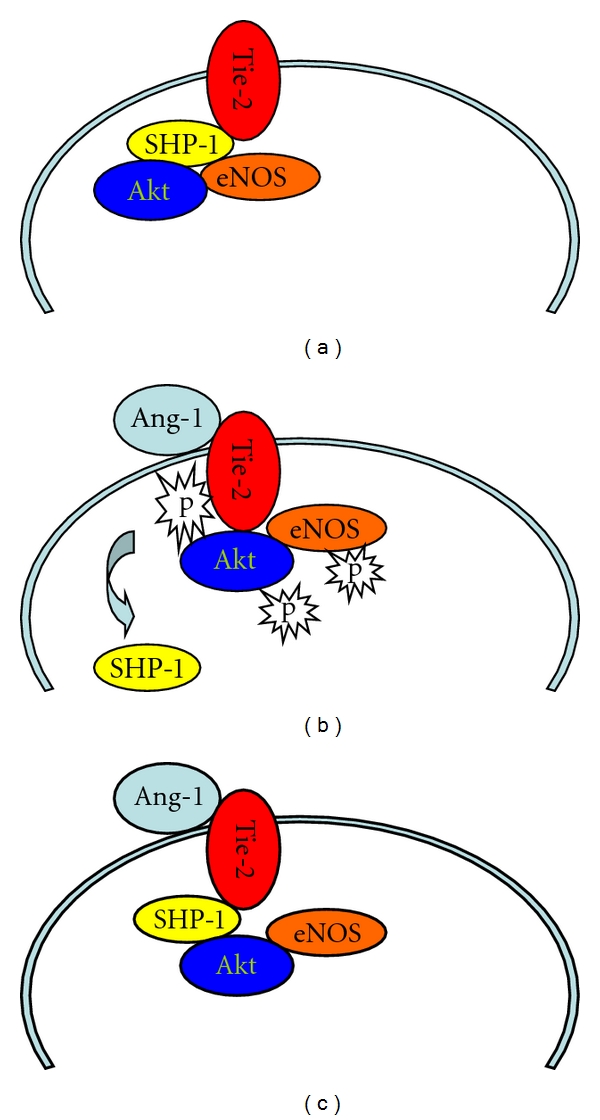
Illustrating our working hypothesis for the SHP-1-induced disruption of Ang-1/Tie-2 signaling under HG conditions and in diabetes. (a) In a resting state, SHP-1 maintains Tie-2 inactivation. (b) Stimulation with Ang-1 causes a dissociation of SHP-1 and Tie-2, thus leading to Tie-2 tyrosine phosphorylation, and its downstream signaling Akt and eNOS activation. (c) Stimulation with Ang-1 fails to lead to the dissociation of SHP-1 and Tie-2, thus resulting in a disruption of Ang-1/Tie-2 signaling under hyperglycemic conditions and in diabetes. NG: normal glucose; HG: high glucose; SHP-1: protein tyrosine phosphatase-1.

 The Src-homology-domain-2- (SH2-) containing tyrosine phosphatases (SHP-1 and SHP-2) have been shown to interact with multiple growth factor receptors including Tie-2 [27, 28]. SHP-2 is primarily associated with enhanced cell growth, whereas SHP-1 has been shown to play a negative regulatory role in endothelial cell proliferation [18, 28–30]. SHP-1 suppresses VEGF and EGF-induced endothelial proliferation, whereas knockdown of SHP-1 augments VEGF- and FGF-2-induced angiogenic responses [20, 31]. SHP-1 also showed to negatively modulate glucose homeostasis via de-phosphorylation of insulin RTK signaling [32]. Our study demonstrated that SHP-1 expression was significantly increased whereas SHP-2 expression remained unchanged in diabetic db/db mouse hearts. Our present study also demonstrated that SHP-1 works as a novel client protein for Tie-2, and stimulation with Ang-1 led to SHP-1 dissociation from Tie-2, implicating a potential interaction between SHP-1 and Ang-1-induced Tie-2 phosphorylation. This notion was further validated by our finding that exposure of MHMEC to HG increased SHP-1/Tie-2 association but decreased Tie-2 phosphorylation. This was consistent with our previous studies that Ang-1-induced Tie-2 phosphorylation was damped under HG conditions [33]. Taken together, the present study reveals a potential novel mechanism for the disruption of Ang-1/Tie-2 signaling by SHP-1 in diabetes. We speculate that protein tyrosine phosphatases, including SHP-1, maintain Tie-2 inactivation by de-phosphorylation, whereas stimulation with Ang-1 leads to dissociation of SHP-1 from Tie-2 and results in Tie-2 phosphorylation and its downstream signaling Akt and eNOS activation. Under hyperglycemic conditions and in diabetes, stimulation with Ang-1 fails to cause the dissociation of SHP-1 from Tie-2, resulting in disruption of Ang-1/Tie-2 signaling (Figure 8).

 Our data also demonstrated that knockdown of SHP-1 by siRNA significantly prevented HG-induced caspase-3 activation and endothelial apoptosis. Our study further demonstrates that inhibition of PTP augmented Ang-1-induced cell survival under HG conditions and restored angiogenic responses in diabetic vessel explants. Inhibition of PTP has been shown to enhance angiogenic signaling and promote VEGF-induced angiogenesis [34]. Inhibition of PTP also promoted collateral blood vessel formation and increased blood flow in a rat model of hind-limb ischemia [14, 15]. Inhibition of PTP has been shown to attenuate endothelial dysfunction via upregulation of eNOS in the mouse model of chronic heart failure [35] and treatment with the nonselective PTP inhibitors such as vanadate and BMOV-enhanced insulin receptor activation and restored insulin signaling in diabetic rats [36–38]. The protective effect of PTP inhibitors on endothelial cell dysfunction was mediated by the enhancement of Akt/eNOS phosphorylation in diabetic rats [36]. Consistent with these findings, our data showed that pretreatment of MHMEC with a PTP inhibitor enhanced Ang-1-induced Akt/eNOS phosphorylation. Our present study also demonstrated that systemic treatment of diabetic db/db mouse with the PTP inhibitor BMOV significantly suppressed SHP-1 expression and increased eNOS expression. This was accompanied by increase in myocardial capillary density. Our study provides new evidence that diabetes may impede angiogenesis by a mechanism involving upregulation of PTP activity which negatively regulates angiogenesis by inhibition of angiogenic growth factor phosphorylation such as Ang-1/Tie-2 system.

### 4.1. Limitation of This Study

 Other PTPs, including PTP1B, SHP-2, PTP-*ε*, VE-PTP, CD148, may also play key roles in the regulation of myocardial angiogenesis in diabetes. Further elucidation of the intracellular mechanisms of PTP with, such as, PTPB1 on diabetes-associated impairment of angiogenic signaling and angiogenesis is needed. We acknowledge that it is technically impossible to examine all PTPs enzymes in a similar manner since specific inhibitors are lacking for each individual isoform of the PTPs. We also acknowledge the potential integrated effects of SHP-1 and PKC beta signaling. Identification of all the mechanisms involved will require additional experiments to evaluate the roles of PTPs and PKC signaling pathways in diabetes-associated impairment of angiogenesis.

In summary, our present study demonstrates that hyperglycemia and diabetes impair angiogenesis by a mechanism involving upregulation of SHP-1 and SHP-1/Tie-2 association. Our study also shows that pharmacological inhibition of PTP or genetic deletion of SHP-1 enhances Ang-1/Tie-2 signaling and improves angiogenesis in diabetes. Our data implicate that restoration of Ang-1/Tie-2 signaling by PTP inhibitors should be considered as a new therapeutic strategy for the treatment or prevention of diabetic impaired angiogenesis.
